# Cancelled elective operations and 28-day breaches in the NHS in England: an interrupted time series analysis of the 2002 penalty policy, 2008 recession, and COVID-19 pandemic (1994–2023)

**DOI:** 10.1016/j.lanepe.2025.101368

**Published:** 2025-07-02

**Authors:** Laura Quinn, Paul Bird, Timothy P. Hofer, Richard Lilford

**Affiliations:** a Department of Applied Health Sciences, University of Birmingham, Birmingham, UK; b NIHR Birmingham Biomedical Research Centre, University Hospitals Birmingham NHS Foundation Trust and University of Birmingham, Birmingham, UK; c Institute for Translational Medicine, University Hospitals Birmingham NHS Foundation Trust, Birmingham, UK; d Center for Clinical Management Research, Veterans Affairs Ann Arbor Health System, Ann Arbor, MI, USA; e Center for Clinical Management Research – an HSR&D Center of Innovation, Veterans Affairs Ann Arbor Healthcare System, Ann Arbor, MI, USA; f Division of General Internal Medicine, Department of Internal Medicine, University of Michigan Medical School, Ann Arbor, MI, USA

**Keywords:** Elective operations, NHS England, Last-minute cancellations

## Abstract

**Background:**

In 2002, the English National Health Service (NHS) introduced financial penalties for hospitals failing to provide elective operations within 28 days of last-minute cancellations. This study investigates the impact of this policy, the 2008 global recession, and the COVID-19 pandemic on cancelled operations and breaches of the 28-day standard.

**Methods:**

We conducted a retrospective observational study using publicly available NHS England data from 1994 to 2023. Interrupted time series analysis assessed changes in cancelled operations and breaches of the 28-day standard across three key periods: pre- and post-2002 policy implementation, post-2008 recession, and post-COVID-19 pandemic. Subgroup analysis by hospital trust A&E department presence on breaches of the 28-day standard was performed.

**Findings:**

Elective admissions nearly doubled over 30 years, rising from just over 1 million per quarter in 1994 (1,054,818) to almost 2 million in 2023 (1,975,508), an 87% increase. Cancellation rates increased leading up to the 2002 policy change but fell rapidly below 1% afterwards and remained stable. The 2008 recession and COVID-19 pandemic did not impact cancellation rates, but did increase breaches of the 28-day standard. Breaches rose before the 2002 policy, dropped rapidly afterwards (−9.6%, 95% CI: −11.2, −9.0), but increased after the recession and notably post-pandemic (13.0%, 95% CI: 4.9%, 21.0%), remaining high and negating earlier gains. Hospitals with A&E departments experienced higher post-pandemic increases in breach rates (12.7%, 95% CI: 10.8, 14.7) compared to those without (0.3%, 95% CI: −3.7, 4.4).

**Interpretation:**

The 2002 policy effectively reduced breaches of the 28-day standard for many years but could not be maintained after the COVID-19 pandemic, when breach rates reached high levels, especially hospitals with A&E departments that could not protect elective beds. Effective targets require sufficient resource capacity and demand management, ignoring such constraints can lead to self-defeating, unjust policies.

**Funding:**

10.13039/501100022244National Institute for Health and Care Research Applied Research Collaboration West Midlands (NIHR200165).

**Research in context**

**Evidence before this study**

We searched PubMed for articles published on the 17th of February 2025, using: ((cancelled OR postponed OR delay) AND (elective OR planned) AND (surgery OR operation∗ OR procedure∗)) AND (“interrupted time series” OR ITS OR “segmented regression” OR “time series analysis”). The 21 results included some studies using interrupted time series models; however, they were primarily focused on the impact of periods where all elective surgery was cancelled during the COVID-19 pandemic. The most relevant study examined the effect of tariff changes for cancelled operations from 2009 to 2011, but its three-year scope contrasts with our study, which examines cancelled operations over the past 30 years.

In 2002, the English National Health Service (NHS) introduced a policy imposing financial penalties when patients did not receive their operation within 28 days of a last-minute cancellation of an elective surgical procedure. This policy aimed to address the issue of last-minute cancellations, which can be distressing for patients and their family, often exacerbating patient symptoms and affecting mental health. External events such as the 2008 global recession and the COVID-19 pandemic have strained NHS resources potentially affecting last-minute cancellations and breaches of the 28-day standard.

**Added value of this study**

This study provides a comprehensive 30-year analysis of elective admissions, cancellation rates, and breaches of the 28-day standard in the English NHS. It examines the impact of the 2002 policy introducing financial penalties for failing to provide operations within 28 days of a last-minute cancellation, as well as the effects of external events such as the 2008 global recession and the COVID-19 pandemic. Our findings reveal that elective admissions nearly doubled over the past 30 years, with cancellation rates initially increasing but reverting to low levels (under 1%) for many years following the 2002 policy change. The COVID-19 pandemic resulted in a significant increase in breaches of the 28-day standard, with rates remaining high and showing wide variation, especially in hospitals with A&E departments.

**Implications of all the available evidence**

The 2002 policy effectively reduced breaches of the 28-day standard for many years but could not be maintained after the COVID-19 pandemic, when breach rates reached high levels, especially hospitals with A&E departments that could not protect elective beds. This reflected the extreme operational pressures on the English NHS during the pandemic, when NHS England, concerned about overwhelming demand, instructed hospitals to cancel all non-urgent elective surgeries and paused reporting on the cancelled operations standard. Our findings suggest targets are most effective in health systems with spare capacity in resources and/or the ability to control the demand for inpatient beds. Ignoring such constraints can result in ineffective or harmful policies.

## Introduction

This study focuses on cancellations of elective surgical procedures in England, specifically those cancelled on the day of admission or within 24 h of the scheduled operation time. These cancellations are classified as ‘last-minute’ cancellations.^1^ Last minute cancellations can occur due to clinical reasons related to the patient, such as the patient being unwell, or for reasons related to lack of capacity, like the unavailability of a ward or Intensive Treatment Unit (ITU) bed, or due to the absence of personnel such as the surgeon, anaesthetist, theatre staff, or surgical equipment or the operating theatre being occupied due to an emergency case.^2^ The cancellations due to lack of capacity are often a reflection of wider pressures on the hospital. Beds cannot be protected indefinitely for any purpose and beds notionally allocated to a particular speciality may be released to another experiencing high demand resulting from increased levels of emergency admissions and or increased length of stay resulting from challenges in discharging patients.

Last-minute cancellations can be extremely disappointing for patients and their families, often exacerbating patient symptoms and affects mental health.^3^ Since patients with the lowest clinical priority tend to be cancelled, they risk not being prioritised for readmission.

Last-minute cancellations are also disruptive for hospital trusts as it can mean precious operating theatre time being unused or under-utilised if the cancelled case cannot be replaced at short notice. To avoid breaching the 28-day standard and incurring financial penalty, hospitals often need to reschedule the cancelled patient promptly, which may require rearranging other patients' procedure dates. However, this can lead to challenges in meeting other performance targets, as prioritising all patients equally reduces flexibility within already tightly schedule theatre lists, making it increasingly difficult to maintain overall operational efficiency.

In order to mitigate the harms and distress to the patient, the UK government mandated that all patients with last-minute cancellations for non-clinical reasons should be prioritised to receive their surgery within 28 days of their original operation date^1^ even if this means using independent hospitals.^4^ Since April 2002, hospitals face financial penalties if they fail to meet this target, as they forgo payments for any patients not treated within the specified timeframe, as well as incurring the cost of undertaking the procedure, or arranging for it to be undertaken in the private sector. In 2019/20, NHS England calculated the average tariff paid for an elective procedure to be £4612,^5^ with costs varying widely between around £1000 and £20,000 for more complex procedures. Highly specialised procedures, such as transplants, can routinely cost over £50,000 but are rarely subject to non-clinical cancellations. Failure to re-schedule and complete the cancelled surgery within 28 days is termed a “breach” of the standard and some or many of these may occur for the same reasons as the original cancellations. Nevertheless, in setting a standard with strong patient level financial incentives the government presumes de facto that hospitals should be able to completely avoid breaches with better prioritisation, planning, and organisation.

Over the past three decades, various external events may have influenced the trends in cancelled operations and breaches of the cancelled operations standard. These factors include financial and operational pressures on the NHS. The global recession in 2008 initiated budget cuts, further straining NHS resources and increasing workload due to rising mental health issues.^6^ The COVID-19 pandemic, beginning in the UK during the last financial quarter of 2019/20, compounded these challenges. In response to the pandemic, NHS England instructed hospitals to cancel all non-urgent elective operations to prioritise care for COVID-19 patients.^7^ During this period, national reporting on cancelled operations and breaches was suspended, resulting in a seven-quarter hiatus in publicly available data from the last quarter of 2019 to the second quarter of 2021.

The presence of a major A&E department is likely to impact hospital capacity, as hospitals with A&E departments must accommodate emergency admissions. This may influence both cancellations and breaches of the 28-day standard, particularly during periods of high demand or due to external events. Therefore, stratifying the analysis by presence of A&E departments allows us to explore whether emergency pressures contribute to elective operation cancellations and breaches.

This study examines the trends of cancelled operations and breaches during and after the 2002 policy change, the financial period of austerity following the 2008 global recession, and the COVID-19 pandemic. Additionally, the study will assess how these effects vary by hospital trusts with and without A&E departments.

## Methods

### Study design and data collection

This study is a retrospective observational study of national elective admissions, last minute cancelled operations and breaches of the 28-day cancelled operations standard across NHS England hospital trusts from the first quarter of 1994 to the third quarter of 2023. An NHS hospital trust is an organisation unit with the English NHS, which manages one or more hospitals or healthcare services within a defined area.

Data are available on the NHS England website and published quarterly (https://www.england.nhs.uk/statistics/statistical-work-areas/cancelled-elective-operations/).^2^ Data on cancelled operations and breaches are nationally mandated and in a standard format but are self-reported by hospital trusts. As mentioned, data was collected or published during the COVID-19 pandemic (from the fourth quarter of 2019 to the second quarter of 2021) after all English hospitals were told to suspend all non-urgent elective surgery from 15th April 2020.

The main analysis uses nationally aggregated data: the number of elective admissions, cancelled operations, and breaches of the 28-day cancelled operations standard per financial quarter. An elective admission is defined as any patient admitted to hospital for a planned procedure, operations cancelled before admission are excluded, as only actual admissions are counted in the denominator. Cancelled operations are those cancelled for non-clinical reasons on the day or within 24 h before the scheduled operation. Breaches of the 28-day cancelled operations standard occur when patients are not re-admitted and treated at NHS expense within 28 days of their original operation date.

For the secondary analysis, hospital trust level data on cancelled operations and breaches of the 28-day standard per financial quarter were available from the first quarter of 2011 to the second quarter of 2023. This data also includes information on the presence of major A&E departments, defined as those with a consultant-led (senior doctor) 24 h service with full resuscitation facilities. Trust level data were aggregated by the presence or absence of a major A&E department.

Results have been reported in line with the Reporting of studies Conducted using Observations Routinely collected Data (RECORD), an extension of the Strengthening the Reporting of Observational Studies in Epidemiology (STROBE) standards.

### Statistical methods

#### Main analysis

The main analysis was conducted using data aggregated at national level across all NHS hospital trusts in England. We visually inspected and summarised data on the national number of elective admissions, cancelled operations and breaches of the 28-day cancelled operations standard over the study period. For each financial quarter, we calculated the national percentage of cancelled operations as a proportion of all elective admissions, and percentage of breaches as a proportion of all last-minute cancelled operations.

We divided the data by key events, including the policy change in the first quarter of 2002, the onset of the global recession in the first quarter of 2008, and the COVID-19 pandemic in the last quarter of 2019. Data were unavailable during the COVID-19 pandemic, we excluded this gap and modelled the post-Covid-19 pandemic as a separate segment to avoid assumptions of continuity across missing data. We summarised data for these specific time periods using medians and interquartile ranges.

To determine shift and trend changes due to interruptions, we fitted interrupted time series models using an Autoregressive Integrated Moving Average (ARIMA) model. The outcomes were nationally aggregated percentage of cancelled operations (out of elective admissions) and the percentage of breaches (out of cancelled operations) per financial quarter. We included indicators for exposure to the interruptions (pre/post interruptions for policy change, recession and COVID-19 pandemic), time indicator (financial quarter), and an interaction between the interruption exposure and time in our models.

We assessed stationarity, defined as a stable mean and variance over time, using the Augmented Dickey–Fuller test and visually inspection of the data. As no evidence of unstable variance was found, a log transformation was not required. Autocorrelation and seasonality were evaluated using Autocorrelation Function (ACF) plots, Partial Autocorrelation Function (PACF) plots, and Ljung–Box tests. When autocorrelation was detected, appropriate autocorrelation terms were incorporated into the models. When seasonality was present, indicators for financial quarters were included as covariates. Model adequacy was further assessed by examining the residuals of the fitted ARIMA models. We checked that residuals were approximately normally distributed, exhibited no remaining autocorrelations, and resembled white noise using ACF plots, PACF plots, and the Ljung–Box test.

As a supplementary analysis, we also fitted logistic regression models were fitted to the outcomes using generalised linear models with a binomial family and logit link, both with and without robust standard errors. These models provided similar trends to those observed in the ARIMA models, supporting the robustness of our findings. Main results are reported from the ARIMA models, as they account for autocorrelation and allow direct interpretation of absolute percentage changes.

#### Secondary analysis

The secondary analysis was performed using data at hospital trust level. To illustrate the variation in breach rates across individual hospital trusts, we graphically displayed the distribution of trust level breach percentages by financial quarter using box plots. For modelling, counts of cancelled operations and breaches were aggregated by hospital trusts with and without a major A&E department. We then calculated the percentage of breaches out of cancelled operations for each group by financial quarter. We then fitted interrupted time series models using ARIMA separately for hospital trusts with and without A&E departments. Our outcome was the percentage of breaches per financial quarter. We also included an indicator for the exposure to the interruption (COVID-19 pandemic), time indicator (financial quarter), and an interaction between the interruption exposure and time in our models. We tested for stationarity and autocorrelation using the same methods as for the main analysis and checked model residuals to confirm model adequacy. Due to evidence of non-normality in residuals for hospital trusts without A&E departments, additional analyses using generalised linear models were performed, results were consistent with the main findings and are provided in the Supplementary Material.

Estimates and 95% confidence intervals were reported for shift and trend changes. All analyses were completed in Stata v18.1.

### Patient and public involvement

Patients or the public were not involved in the design, or conduct, or reporting, or dissemination plans of our research.

### Ethics approval

This study used only publicly available, aggregated data and did not require ethical approval.

### Role of the funding source

This study was funded by National Institute for Health and Care Research (NIHR) Applied Research Collaboration (ARC) West Midlands (NIHR200165) and had no role in the study design, data collection and analysis, interpretations of data, preparation of the manuscript, or decision to publish.

## Results

### Summary of elective admissions, cancelled operations, and breaches

**Fig. 1:**
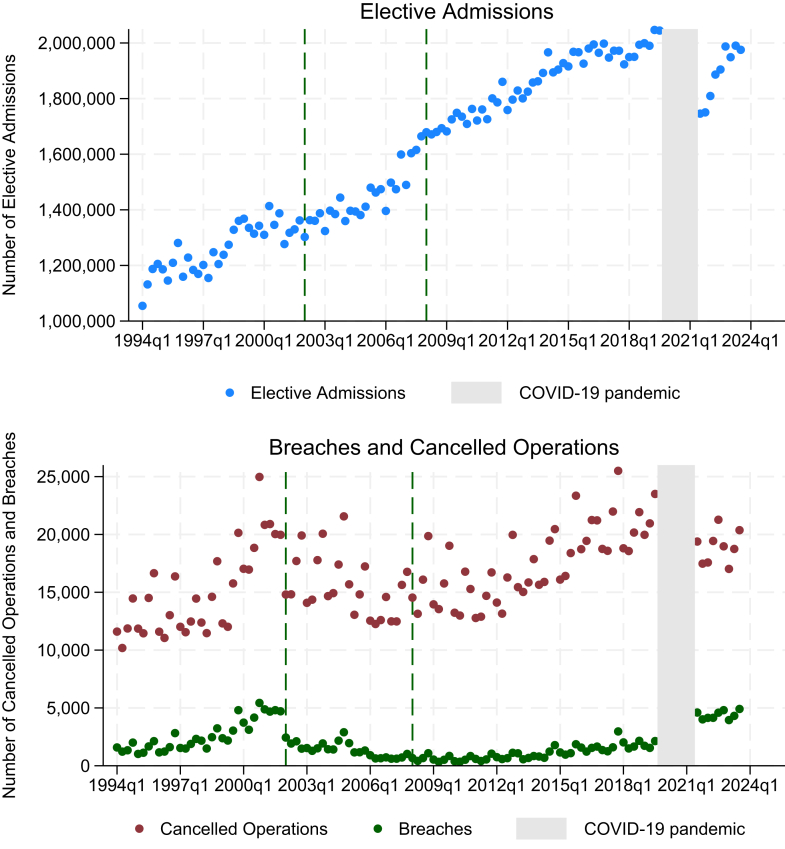
National number of elective admission, cancelled operations and breaches across NHS England hospital trusts per financial quarter from 1994 to 2023. Dots represent data, dashed lines represent start of 2002 policy change and the 2008 global recession. Grey area represents COVID-19 pandemic when data for cancelled operations was not collected.

In NHS England, from 1994 to 2023, there were 179,484,516 elective admissions for surgery or medical procedures. Due to national reporting datasets, we are unable to isolate this to solely elective surgical admissions. Of these admissions, 1,847,855 (1.03%) resulted in cancelled operations, and 208,384 (11.28%) of those breached the standard for completion within 28-days of the original cancelled operation date. Elective admissions have been increasing over time, with the number of elective admissions per financial quarter almost doubling in the last 30 years from one to two million [Fig. 1]. The target is derived from all elective admissions, not elective surgical admissions. As the data is nationally aggregated, we are unable to separate these out. The lowest number of elective admissions occurred in the first quarter of 1994 (1,054,818) and the highest the second quarter of 2019 (2,046,888) before the COVID-19 pandemic [Supplementary Material].

Visually in Fig. 1, the number of cancelled operations and breaches increased from 1994 to 2002. Following the introduction of the 2002 policy both these quantities dropped, most precipitously in the case of breaches. During the early years of the recession, the number of cancelled operations and breaches remained reasonably stable. However, in the years immediately preceding the COVID-19 pandemic, both started to increase. After the pandemic, there were fewer cancelled operations, but more breaches of the 28-day cancelled operations standard.

The average percentage of cancelled operations per elective admissions, and breaches per cancelled operation are summarised by time period in Table 1.

**Table 1:** Percentage of operations cancelled at the last minute and breaches of the 28-day cancelled operation standard by time period.

Time period	Number of quarters	Cancelled operations		Breaches	
		Cancelled operations/elective admissions (%)	Median percentage (IQR)	Number of breaches/cancelled operations (%)	Median percentage (IQR)
Pre-policy change	32	481,117/40,257,840 (1.20)	1.15 (1.00–1.35)	83,542/481,117 (17.36)	16.50 (12.59–20.57)
Post-policy change	24	372,316/34,663,150 (1.07)	1.07 (0.91–1.21)	33,676/372,316 (9.05)	8.68 (5.65–11.43)
Post-recession	47	824,144/87,564,004 (0.94)	0.94 (0.83–1.02)	51,664/824,144 (6.27)	5.63 (4.15–7.39)
Post-pandemic	16	170,278/16,999,522 (1.00)	1.00 (0.95–1.03)	39,502/170,278 (23.20)	23.23 (22.97–23.75)
Overall	119	1,847,855/179,484,516 (1.03)	1.00 (0.90–1.13)	208,384/1,847,855 (11.28)	8.81 (5.50–14.51)

Note: Percentages are higher for breaches than cancelled operations (they are different denominators) but the number of breaches is much lower than the number of cancelled operations (Fig. 1).

Data collection: Data not collected from 2019q4 to 2021q2 due to the COVID-19 pandemic.

Time period definitions: Pre-policy change: (1994q1–2001q4), post-policy change (2002q1–2007q4), post-recession (2008q1–2019q3), post-pandemic (2021q3–2023q3).

### Interrupted time series analysis of cancelled operations and breaches

Both interrupted time series models included an AR^1^ process to account for autocorrelation and incorporated indicators for financial quarters to adjust for seasonality.

#### Cancelled operations

**Fig. 2:**
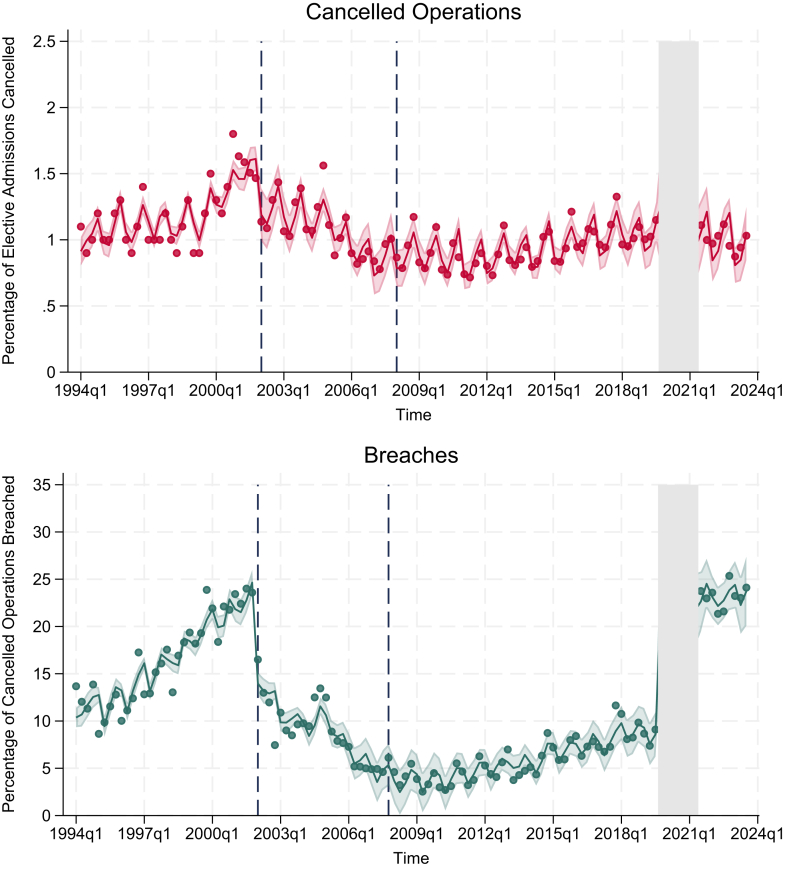
Interrupted time series of national cancelled operations and breaches. Interruptions for 2002 policy change, 2008 recession, and COVID-19 pandemic. Top figure displays percentage of cancelled operations out of elective admission. Bottom figure displays percentage of breaches out of cancelled operations. Dots represent data, lines represent model prediction, and shaded area represents 95% confidence intervals around model predictions. Dashed lines represent start of 2002 policy change and the 2008 global recession. Grey area represents COVID-19 pandemic when data for cancelled operations was not collected.

Nationally, cancelled operations were increasing by 0.01% per financial quarter (95% CI: 0.01–0.02) before the 2002 policy change. When the policy was introduced, there was no immediate significant change in the percentage of cancelled operations (−0.10%, 95% CI: −0.31 to 0.10). However, this was followed by a decrease of 0.02% per financial quarter (95% CI: −0.04 to −0.01). There was no evidence of a change in percentage of cancelled operations at the start of the 2008 recession or the COVID-19 pandemic. There was also no evidence of a trend post-recession or pandemic, and no evidence of a change in trend from pre- to post-pandemic (Table 2/Fig. 2).

**Table 2:** Interrupted time series models for cancelled operations and breaches of the 28-day cancelled operation standard.

Time period	Cancelled operations	Breaches
	Percentage point change (95% CI)	Percentage point change (95% CI)
Pre-policy change	0.01 (0.01–0.02)	0.42 (0.35–0.48)
Shift change at policy (2002)	−0.10 (−0.31 to 0.10)	−9.60 (−11.17 to −8.03)
Post-policy change trend	−0.02 (−0.04 to −0.01)	−0.41 (−0.54 to −0.28)
Shift change at recession (2008)	0.04 (−0.16 to 0.23)	−0.97 (−4.10 to 2.16)
Post- recession trend	0.00 (−0.00 to 0.01)	0.13 (0.05–0.22)
Shift change at pandemic (2019)	0.03 (−0.52 to 0.57)	12.95 (4.87–21.03)
Post-pandemic trend	−0.01 (−0.05 to 0.04)	0.11 (−0.59 to 1.82)
Change in trend from pre to post pandemic	−0.01 (−0.06 to 0.03)	−0.02 (−0.73 to 0.69)

All estimates are percentage point changes per financial quarter.

#### Breaches of 28-day cancelled operations standard

Before the introduction of the 2002 policy change, breaches of the 28-day cancelled operations standard increased by 0.42% per financial quarter (95% CI: 0.35–0.48). Upon the policy's implementation, there was a reduction in breaches of −9.60% (95% CI: −11.17 to −8.03). Following the policy change, breaches decreased by 0.41% per financial quarter (95% CI: −0.54 to −0.28). There was no evidence of a change in breaches at the onset of the 2008 recession, however, breaches increased by 0.13% per financial quarter afterwards (95% CI: 0.05–0.22). After the COVID-19 pandemic, breaches increased by 12.95% (95% CI: 4.87–21.03). Following the pandemic, the breach rate remained high with no evidence of a trend (0.11, 95% CI: −0.59 to 1.82). There was also no evidence of a change in trend of breaches from pre- to post-pandemic (−0.02, 95% CI: −0.73 to 0.69) (Table 2/Fig. 2).

### Interrupted time series of breaches by presence of A&E departments

**Fig. 3:**
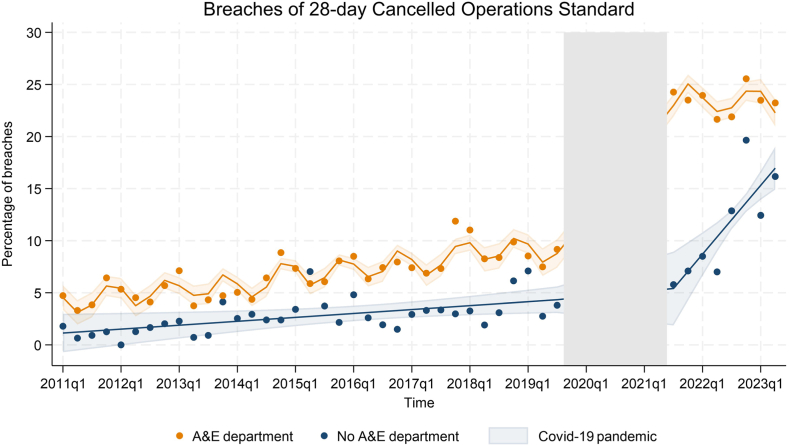
Interrupted time series of aggregated cancelled operations and breaches in NHS hospital trusts, by presence of A&E department. Data are aggregated by financial quarter into two groups: hospital trusts with an A&E department (orange) and hospital trusts without an A&E department (blue). The grey area indicates the period during COVID-19 pandemic when data on cancelled operations were not collected.

The interrupted time series model for hospital trusts with A&E departments included an AR^1^ process to account for autocorrelation and indicators for financial quarters to adjust for seasonality. For non-A&E hospital trusts, residual diagnostics showed no evidence of autocorrelation or seasonality, so neither AR^1^ nor seasonal terms were included. For hospital trusts with A&E departments, the percentage of breaches increased by 0.16% per financial quarter pre-pandemic (95% CI: 0.11–0.22). During the pandemic, the percentage of breaches increased by 12.74% (95% CI: 10.80–14.68). There was no evidence of a trend in breaches after the pandemic (0.08, 95% CI: −0.06 to 0.23), nor was there evidence of a change in trends before and after the COVID-19 pandemic (−0.08, 95% CI: −0.23 to 0.07) (Table 3/Fig. 3).

**Table 3:** Interrupted time series models of breaches of the 28-day cancelled operation standard by presence of A&E department.

Time period	A&E department	No A&E department
	Percentage point change (95% CI)	Estimate percentage point change (95% CI)
Pre-pandemic trend	0.16 (0.11–0.22)	0.09 (0.01–0.17)
Shift change at pandemic	12.74 (10.80–14.68)	0.32 (−3.73 to 4.37)
Post-pandemic trend	0.08 (−0.06 to 0.23)	0.87 (0.49–1.26)
Change in trend from pre to post pandemic	−0.08 (−0.23 to 0.07)	0.78 (0.40–1.16)

All estimates are percentage point changes per financial quarter.

For hospital trusts without an A&E department, breaches increased by 0.09% per financial quarter before the COVID-19 pandemic. During the COVID-19 pandemic, there was no evidence of a shift change (0.32, 95% CI: −3.73 to 4.37). Since the COVID-19 pandemic, breaches have increased by 0.87% per financial quarter (95% CI: −0.49 to 1.26). There is evidence of an increase in the trend from pre- to post-pandemic of 0.78% (95% CI: 0.40–0.16).

### Distribution of breaches by presence of A&E department

**Fig. 4:**
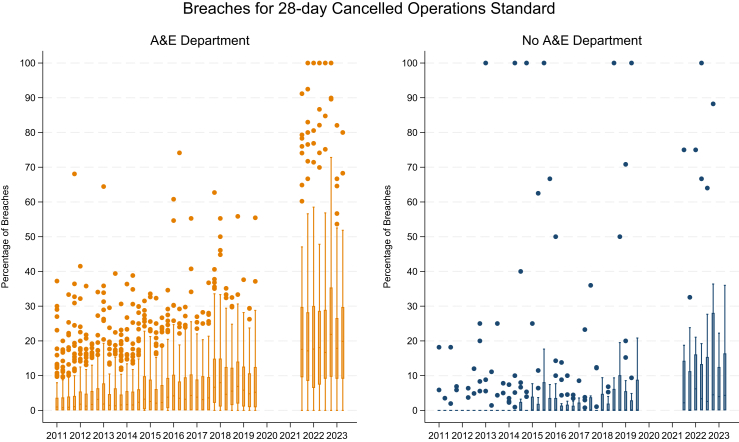
Box plot of breaches for 28-day cancelled operations standard at NHS hospital trust level, by presence of A&E department. Box plots show the distribution of breaches across hospital trusts with an A&E department (orange) and without an A&E department (blue) by financial quarter from 2011 to 2023. The dashes from 2011 to 2014 in hospitals without an A&E department indicate box plots where values up to the whiskers are zero and only outlying trusts are shown. The blank area indicates the period during COVID-19 pandemic when data for cancelled operations were not collected.

For hospital trusts with A&E departments, the average percentage of breaches across hospital trusts increased from 1% to 5% over the 9 years leading to the pandemic, and variation between hospital trusts also increased over this period. After the COVID-19 pandemic, the average percentage of breaches increased from 17% to 21% and again the variation across hospital trusts also increased. Some of the increase in variation is expected as a statistical property of proportions, since variance naturally rises as the proportion increases (Fig. 4).

Before the COVID-19 pandemic, the average percentage of breaches across hospital trusts without A&E departments was 0%. The variation in breaches across hospital trusts started to increase from 2015. After the COVID-19 pandemic, the average percentage and variation increased but was still much lower than hospital trusts with A&E departments.

## Discussion

Elective admissions have nearly doubled over the last 30 years (Fig. 1). In the years leading up to the 2002 policy, there was an increase in cancelled operations, but this reverted to the previously low levels (less than 1% of elective operations) following the introduction of the policy despite an increase in admissions. The 2002 policy aimed to reduce harm from last-minute cancellations by ensuring patients were rescheduled to have their operation within 28 days, rather than reducing cancellations themselves. The 2008 recession and COVID-19 pandemic had no impact on cancellation rates. As with cancellations, breaches of the 28-day cancelled operations standard also increased before the 2002 policy change. Within a year of the policy change, there was a large drop in breaches followed by an ongoing gradual drop thereafter until 2010. There was no immediate change in breaches when the recession started in 2008, but a gradual upward trend then ensued. This suggests that the financial pressures following the recession accumulated gradually. In the setting of massive COVID-19 related waiting list growth, breaches increased markedly after the pandemic and have remained at this high level.

The combination of an unremitting increase in the level of elective activity with an inflation adjusted reduction of hospital budgets should be expected to produce a corresponding reduction in the resilience of hospitals. From 2010 to 2020 there has been a reduction in the ability of hospitals to meet national targets generally^8^, ^9^, ^10^ and the growth of elective waiting lists, with demand for inpatient beds consistently outstripping capacity post-COVID-19.^8^ Given long waiting lists, both clinicians and managers alike are likely to push the occupancy limits to maximise elective throughput. A system operating at or beyond occupancy limits will clearly have a greater the likelihood of cancellations.

Hospitals with A&E departments differed from those without A&E departments even before the pandemic. While hospitals without A&E departments experience cancelled operations, they have virtually no breaches of the 28-day standard. This could be attributed to the fact that they can plan and control demand on beds—a theory supported by the data, since it is the hospitals with an A&E that experience seasonal effects with a rise in breaches each winter. After the pandemic however, even the hospitals without an A&E experience a rise in breaches. This rise is much smaller rise than observed in hospitals with an A&E, at least in absolute terms; about five percent versus twenty percent.

The existing literature on NHS cancelled operations and breaches of cancelled operations standard are limited. The available studies primarily consist of descriptive articles that track changes in cancelled operations and breaches over time.^11^, ^12^, ^13^ Some single-site studies describe the reasons for cancellations. Additionally, one study examined factors associated with cancelled operations over a one-week period across 245 NHS hospitals.^14^ However, there is a lack of analytical studies that investigate changes in cancelled operations and breaches over time, particularly in relation to whether these vary by hospital trusts with and without A&E departments. It is important to clarify the primary intention of the 2002 policy was not to decrease the overall number of cancellations, but to minimise the negative impact on patient by guaranteeing a rescheduled operation within 28 days of cancellation.

Drawing on literature from Walter Shewhart and others,^15^^,^^16^ reduction in variation is a key objective of quality improvement. Conversely, widening variation suggests a system that is ‘under stress’ or not in ‘control’ even if the precise mechanism cannot be identified. The effect of having an A&E department is informative in this regard; clearly the presence of an A&E makes it harder to control demand for beds and help deal with the ‘stress’ of surges in activity. This is evident in breaches of the 28-day standard in England over the post-COVID-19 era, where the system has been under great stress with increasing waiting lists and waiting times. The increased variation observed is suggestive of a health system which is struggling to cope with operational pressures.^17^ It should be recognised that part of the observed increase in variation arises naturally because the variability of a proportion tends to grow as its level rises. However, the persistent and widening differences between hospital trusts, especially those with A&E departments, indicate disparities in performance under stress. Hospitals without an A&E are only partially protected since not only do they experience a rise in breaches as stated above, but variance among this group also widens, suggesting again that they are not sequestrated from the stress affecting the system as a whole. The precise mechanism behind the increase in hospital trusts without A&E departments is unclear and may vary from one place to another. It remains to be seen whether, over time, hospitals both with and without A&E departments will revert to their pre-pandemic performance.

For hospitals with A&E departments, breaches have always been higher suggesting that emergency admissions and seasonal ‘winter pressures’ affect the level of breaches. The ‘winter pressures’ refer to the increased A&E attendances and admissions from January to March due to an increase in influenza, norovirus, respiratory exacerbations and to a lesser extent trauma resulting from slips, trips and falls brought on by cold or icy weather. The theory of ‘protecting’ bed capacity from emergency admissions to maximise efficient throughput of elective operations began to gain traction in the early 2000s, with the creation of day case or 23 h stay beds, often only open Monday–Friday. By not having these beds open full time, this precluded emergency admissions from being admitted to them. This concept has been further reinforced using private sector provided beds (where there is no A&E department) in order to provide additional protected capacity to tackle the waiting list backlog from both pre- and post-pandemic periods. This led to a drive for High Volume Low Complexity (HVLC) centres linked to the Getting It Right First Time (GIRFT) programme in the NHS.^18^ Alongside this, some hospital trusts with multiple sites have sought to re-arrange their services to create ‘cold elective sites’^19^ where there is no A&E department. This is on the basis that the requirement to physically transfer patients over some distance will deter sites with A&E departments from using these beds for emergency admissions. However, there is debate on this point, with some arguing co-location is more productive, providing elective capacity can be maintained on a mixed site.^20^ Although hospital trusts develop tailored strategies annually to guard against ‘winter pressures’, if breaches of the 28-day cancelled operations standard are taken as a marker of effectiveness, there is still significant disruption caused by seasonality and more work needs to be done to reduce variation particularly post-COVID-19. It is also concerning from a policy makers perspective to see hospitals without A&E departments who have historically been able to meet the target with low levels of variation now breaching the target post-pandemic with much higher levels of variation.

If this analysis had been done in 2010 then the findings would have been strongly supportive of this financial incentive-based policy. However, looking at the subsequent decade a concerning trend of gradually increasing breaches was noted in the setting of increasing demand and reduced budgets. In the current post-pandemic epoch, the rate of breaches at 20 percent is almost what it was in the immediate run-up to implementation of the policy in 2002. These findings would support the conclusion that the policy worked under conditions and constraints present at the time it was instituted and over the ensuing 10 years but that there will be a level of system stress where the policy loses traction. That ‘stress’ we must assume reflects the interaction of supply and demand for beds; the point is reached where, perhaps quite suddenly, human and physical resources cannot cope with the demand even if those resources are optimally managed.

When a system reaches a ‘breaking point’ imposing fines can only harm the institutions that are most stressed. There is no precise or proven method to make the ‘diagnosis’ that the point has been reached where the incentive does more harm than good. However, policy makers should be alert to the danger. We would be surprised if this particular incentive is doing any good and if not, it is likely doing at least some harm. In our opinion it is time to ‘rest’ this incentive. Such an approach has already been taken with regard to the A&E 4 h target in England, which was reduced from 98% to 95% in 2010 (pre-COVID-19), and then reduced further to 78% post-COVID-19 in recognition of the fact this was largely unobtainable for organisations.^21^ More important is the general point—policy makers should be vigilant and be prepared to suspend or retire incentives as circumstances change—there is no ‘incentive for all seasons’.

The target is self-reported by hospital trusts against a national definition and according to prescribed timeframes. However, it is not systematically or rigorously independently audited which could potentially leave it open to being manipulated by provider organisations. Whilst an organisational incentive could exist for hospital administrators to report cancellations as clinical rather than non-clinical to avoid sanction, there is a similar individual incentive for surgeons to report and highlight capacity constraints in order to support future service planning, provision and business case development. Whilst we cannot exclude the possibility of gaming by hospital trusts to achieve the standard,^9^^,^^20^^,^ and there is some limited evidence of this in relation to other targets,^10^^,^^22^ we do not believe this to be a significant factor in relation to this target. As the target is self-reported we cannot make any objective judgement as to whether data capture, data quality or validity has changed over the study period, or as to any variation between geographic areas or sizes of hospital trusts.

The strengths of this study include the availability of data for all hospital trusts in the NHS in England over an extended time period and the use of interrupted time series analysis, which allows for the measurement of changes due to interruptions when randomised trials are not possible. However, there are several limitations. The analysis uses aggregated data by financial quarter, which does not account for variation between individual national trusts. Data on elective admissions were not available at trust level, limiting the ability to analyse cancelled operations rates by hospital trusts with and without major A&E departments. There are only a small number of NHS hospital trusts which do not have a major A&E department and so the sample for this subgroup analysis is small. All data for the secondary analysis is provided at hospital trust level, and there are hospital trusts that operate across multiple sites, only some of which have major A&E departments. In the NHS, major A&E departments are consultant-led (senior doctor-led) 24-h services equipped to handle serious emergencies, while minor A&E department are typically doctor-led or nurse-led services that treat less severe conditions with fewer resources. Due to the nature of the aggregate data, we cannot control for this, but the implication is that the effects we observed might be slightly depressed. Future work could include a more detailed analysis comparing outcomes between major and minor A&E departments, as well as looking at factors associated with cancellation rates and breaches at hospital trust level, such as hospital size and occupancy rates.

The results of this study suggest that the 2002 policy change effectively reduced breaches of the 28-day cancelled operation standard, however, that breaches began to rise again, indicating long-term effectiveness may wane. Hospitals with A&E departments also had higher breaches, suggesting that emergency admissions and seasonal pressures also increase breach rates. The COVID-19 pandemic increased breach rates in hospital trusts, regardless of A&E department presence, suggesting increased pressure. Overall, these findings indicate that reforms are needed to enhance capacity to meet the rising demand in the NHS.

## Contributors

RL, PB, and LQ conceived and designed the study. LQ analysed the data, accessed and verified the underlying data, and wrote the first draft of the manuscript. PB also accessed and verified the data. LQ, PB, TPH, and RL interpreted the findings, revised the manuscript, and had final approval of the submitted paper. RL had final responsibility for the decision to submit the manuscript.

## Data sharing statement

Data are available on the NHS England website and published quarterly. (https://www.england.nhs.uk/statistics/statistical-work-areas/cancelled-elective-operations/).

## Declaration of interests

All authors have completed the ICMJE disclosure forms and declare there are no competing interests.
