# Supplementary material for: Cancelled elective operations and 28-day breaches in the NHS in England: an interrupted time series analysis of the 2002 penalty policy, 2008 recession, and COVID-19 pandemic (1994–2023)
Source: Lancet Reg Health Eur. 2025 Jul 2;56:101368. doi: 10.1016/j.lanepe.2025.101368 (PMC12270809; doi:10.1016/j.lanepe.2025.101368)
Supplement: Supplementary Materials [file mmc1.docx]

### **Supplementary Material 1.** Elective admissions, cancelled operations and breaches of the 28-day cancelled operation standard.

| **Time** | **Elective Admissions** | **Cancelled Operations** | **Breaches** |
| --- | --- | --- | --- |
| 1994q1 | 1054818 | 11603 (1.1) | 1587 (13.68) |
| 1994q2 | 1131778 | 10186 (0.9) | 1226 (12.04) |
| 1994q3 | 1187400 | 11874 (1.0) | 1343 (11.31) |
| 1994q4 | 1205500 | 14466 (1.2) | 2005 (13.86) |
| 1995q1 | 1186000 | 11860 (1.0) | 1025 (8.64) |
| 1995q2 | 1145700 | 11457 (1.0) | 1129 (9.85) |
| 1995q3 | 1209500 | 14514 (1.2) | 1675 (11.54) |
| 1995q4 | 1280923 | 16652 (1.3) | 2130 (12.79) |
| 1996q1 | 1159500 | 11595 (1.0) | 1162 (10.02) |
| 1996q2 | 1228333 | 11055 (0.9) | 1229 (11.12) |
| 1996q3 | 1183909 | 13023 (1.1) | 1614 (12.39) |
| 1996q4 | 1169571 | 16374 (1.4) | 2825 (17.25) |
| 1997q1 | 1201900 | 12019 (1.0) | 1541 (12.82) |
| 1997q2 | 1155000 | 11550 (1.0) | 1492 (12.92) |
| 1997q3 | 1247600 | 12476 (1.0) | 1891 (15.16) |
| 1997q4 | 1205000 | 14460 (1.2) | 2326 (16.09) |
| 1998q1 | 1238400 | 12384 (1.0) | 2174 (17.55) |
| 1998q2 | 1274444 | 11470 (0.9) | 1495 (13.03) |
| 1998q3 | 1328273 | 14611 (1.1) | 2472 (16.92) |
| 1998q4 | 1360385 | 17685 (1.3) | 3239 (18.31) |
| 1999q1 | 1368111 | 12313 (0.9) | 2384 (19.36) |
| 1999q2 | 1335222 | 12017 (0.9) | 2186 (18.19) |
| 1999q3 | 1314083 | 15769 (1.2) | 3042 (19.29) |
| 1999q4 | 1342867 | 20143 (1.5) | 4809 (23.87) |
| 2000q1 | 1310231 | 17033 (1.3) | 3733 (21.92) |
| 2000q2 | 1413750 | 16965 (1.2) | 3116 (18.37) |
| 2000q3 | 1346000 | 18844 (1.4) | 4168 (22.12) |
| 2000q4 | 1387556 | 24976 (1.8) | 5437 (21.77) |
| 2001q1 | 1276916 | 20833 (1.63) | 4881 (23.43) |
| 2001q2 | 1317447 | 20901 (1.59) | 4684 (22.41) |
| 2001q3 | 1329843 | 20036 (1.51) | 4810 (24.01) |
| 2001q4 | 1361880 | 19973 (1.47) | 4712 (23.59) |
| 2002q1 | 1302360 | 14808 (1.14) | 2443 (16.5) |
| 2002q2 | 1362892 | 14826 (1.09) | 1926 (12.99) |
| 2002q3 | 1360623 | 17706 (1.3) | 2119 (11.97) |
| 2002q4 | 1387763 | 19914 (1.43) | 1484 (7.45) |
| 2003q1 | 1323799 | 14092 (1.06) | 1534 (10.89) |
| 2003q2 | 1397181 | 14364 (1.03) | 1293 (9) |
| 2003q3 | 1384664 | 17782 (1.28) | 1509 (8.49) |
| 2003q4 | 1444093 | 20065 (1.39) | 1934 (9.64) |
| 2004q1 | 1359813 | 14672 (1.08) | 1432 (9.76) |
| 2004q2 | 1396681 | 14929 (1.07) | 1412 (9.46) |
| 2004q3 | 1394126 | 17402 (1.25) | 2175 (12.5) |
| 2004q4 | 1381096 | 21566 (1.56) | 2901 (13.45) |
| 2005q1 | 1411379 | 15690 (1.11) | 1959 (12.49) |
| 2005q2 | 1479813 | 13058 (0.88) | 1160 (8.88) |
| 2005q3 | 1462215 | 14819 (1.01) | 1170 (7.9) |
| 2005q4 | 1474246 | 17236 (1.17) | 1322 (7.67) |
| 2006q1 | 1396060 | 12548 (0.9) | 912 (7.27) |
| 2006q2 | 1497881 | 12259 (0.82) | 637 (5.2) |
| 2006q3 | 1474106 | 12600 (0.85) | 652 (5.17) |
| 2006q4 | 1598966 | 14598 (0.91) | 729 (4.99) |
| 2007q1 | 1489633 | 12489 (0.84) | 613 (4.91) |
| 2007q2 | 1603648 | 12482 (0.78) | 615 (4.93) |
| 2007q3 | 1615630 | 15640 (0.97) | 720 (4.6) |
| 2007q4 | 1664482 | 16771 (1.01) | 1025 (6.11) |
| 2008q1 | 1679188 | 14543 (0.87) | 669 (4.6) |
| 2008q2 | 1671318 | 13144 (0.79) | 424 (3.23) |
| 2008q3 | 1680016 | 16094 (0.96) | 668 (4.15) |
| 2008q4 | 1693637 | 19863 (1.17) | 1088 (5.48) |
| 2009q1 | 1682180 | 13958 (0.83) | 539 (3.86) |
| 2009q2 | 1725378 | 13547 (0.79) | 343 (2.53) |
| 2009q3 | 1748999 | 15765 (0.9) | 521 (3.3) |
| 2009q4 | 1735117 | 19026 (1.1) | 855 (4.49) |
| 2010q1 | 1708984 | 13233 (0.77) | 396 (2.99) |
| 2010q2 | 1762988 | 12991 (0.74) | 351 (2.7) |
| 2010q3 | 1721273 | 16784 (0.98) | 523 (3.12) |
| 2010q4 | 1760996 | 15287 (0.87) | 844 (5.52) |
| 2011q1 | 1725958 | 12780 (0.74) | 594 (4.65) |
| 2011q2 | 1800973 | 12892 (0.72) | 415 (3.22) |
| 2011q3 | 1786454 | 14696 (0.82) | 553 (3.76) |
| 2011q4 | 1860415 | 16719 (0.9) | 1048 (6.27) |
| 2012q1 | 1758961 | 14113 (0.8) | 746 (5.29) |
| 2012q2 | 1796340 | 13155 (0.73) | 582 (4.42) |
| 2012q3 | 1829133 | 16281 (0.89) | 663 (4.07) |
| 2012q4 | 1800760 | 19968 (1.11) | 1124 (5.63) |
| 2013q1 | 1825104 | 15443 (0.85) | 1079 (6.99) |
| 2013q2 | 1857919 | 15032 (0.81) | 564 (3.75) |
| 2013q3 | 1862289 | 15852 (0.85) | 679 (4.28) |
| 2013q4 | 1892502 | 17868 (0.94) | 845 (4.73) |
| 2014q1 | 1966435 | 15650 (0.8) | 802 (5.12) |
| 2014q2 | 1894377 | 15898 (0.84) | 691 (4.35) |
| 2014q3 | 1904502 | 19470 (1.02) | 1233 (6.33) |
| 2014q4 | 1927719 | 20464 (1.06) | 1787 (8.73) |
| 2015q1 | 1915905 | 16099 (0.84) | 1157 (7.19) |
| 2015q2 | 1968406 | 16414 (0.83) | 965 (5.88) |
| 2015q3 | 1966783 | 18393 (0.94) | 1092 (5.94) |
| 2015q4 | 1925765 | 23352 (1.21) | 1864 (7.98) |
| 2016q1 | 1980104 | 18730 (0.95) | 1575 (8.41) |
| 2016q2 | 1994782 | 19446 (0.97) | 1228 (6.31) |
| 2016q3 | 1964809 | 21247 (1.08) | 1550 (7.3) |
| 2016q4 | 1997774 | 21219 (1.06) | 1663 (7.84) |
| 2017q1 | 1947534 | 18752 (0.96) | 1359 (7.25) |
| 2017q2 | 1972311 | 18587 (0.94) | 1255 (6.75) |
| 2017q3 | 1972168 | 21984 (1.11) | 1599 (7.27) |
| 2017q4 | 1923248 | 25502 (1.33) | 2970 (11.65) |
| 2018q1 | 1949452 | 18805 (0.96) | 2022 (10.75) |
| 2018q2 | 1950293 | 18568 (0.95) | 1501 (8.08) |
| 2018q3 | 1993676 | 20166 (1.01) | 1665 (8.26) |
| 2018q4 | 1999288 | 21931 (1.1) | 2157 (9.84) |
| 2019q1 | 1990164 | 19969 (1.0) | 1730 (8.66) |
| 2019q2 | 2046888 | 20961 (1.02) | 1548 (7.39) |
| 2019q3 | 2044739 | 23503 (1.15) | 2138 (9.1) |
| 2021q3 | 1745973 | 19390 (1.11) | 4605 (23.75) |
| 2021q4 | 1750434 | 17477 (1.0) | 4015 (22.97) |
| 2022q1 | 1809468 | 17579 (0.97) | 4145 (23.58) |
| 2022q2 | 1886462 | 19439 (1.03) | 4149 (21.34) |
| 2022q3 | 1904705 | 21273 (1.12) | 4593 (21.59) |
| 2022q4 | 1987581 | 18975 (0.95) | 4811 (25.35) |
| 2023q1 | 1948942 | 17024 (0.87) | 3954 (23.23) |
| 2023q2 | 1990449 | 18749 (0.94) | 4317 (23.03) |
| 2023q3 | 1975508 | 20372 (1.03) | 4913 (24.12) |

### **Supplementary Material 2.**

### Interrupted time series models for cancelled operations and breaches of the 28-day cancelled operation standard using Logistic Regression

|  | **Cancelled Operations** | **Breaches** |
| --- | --- | --- |
| **Time period** | **Odds Ratio**  **(95% CI)** | **Odds Ratio**  **(95% CI)** |
| Pre-policy change | 1.01 (1.01 to 1.01) | 1.03 (1.03 to 1.03) |
| Shift change at policy (2002) | 0.91 (0.90 to 0.92) | 0.46 (0.45 to 0.48) |
| Post-policy change trend | 0.98 (0.98 to 0.98) | 0.96 (0.96 to 0.96) |
| Shift change at recession (2008) | 0.97 (0.96 to 0.98) | 0.63 (0.61 to 0.66) |
| Post-recession trend | 1.01 (1.01 to 1.01) | 1.02 (1.02 to 1.03) |
| Shift change at pandemic (2019) | 0.98 (0.96 to 1.01) | 2.43 (2.31 to 2.57) |
| Post-pandemic trend | 1.00 (0.99 to 1.00) | 1.01 (1.01 to 1.02) |
| Change in trend from pre to post pandemic | 0.99 (0.99 to 0.99) | 0.99 (0.99 to 0.99) |

### Interrupted time series models for cancelled operations and breaches of the 28-day cancelled operation standard using Logistic Regression with robust standard errors

|  | **Cancelled Operations** | **Breaches** |
| --- | --- | --- |
| **Time period** | **Odds Ratio**  **(95% CI)** | **Odds Ratio**  **(95% CI)** |
| Pre-policy change | 1.01 (1.00 to 1.02) | 1.03 (1.03 to 1.04) |
| Shift change at policy (2002) | 0.91 (0.81 to 1.03) | 0.46 (0.37 to 0.58) |
| Post-policy change trend | 0.98 (0.98 to 0.98) | 0.96 (0.94 to 0.97) |
| Shift change at recession (2008) | 0.97 (0.89 to 1.06) | 0.63 (0.50 to 0.80) |
| Post-recession trend | 1.01 (1.00 to 1.01) | 1.02 (1.02 to 1.03) |
| Shift change at pandemic (2019) | 0.98 (0.77 to 1.26) | 2.43 (1.91 to 3.10) |
| Post-pandemic trend | 1.00 (0.98 to 1.02) | 1.01 (0.99 to 1.03) |
| Change in trend from pre to post pandemic | 0.99 (0.97 to 1.01) | 0.99 (0.97 to 1.01) |

### **Supplementary Material 3.**

### Interrupted time series models of Breaches of the 28-day Cancelled Operation Standard by presence of A&E department using Logistic Regression

|  | **A&E department** | **No A&E department** |
| --- | --- | --- |
| **Time period** | **Odds Ratio**  **(95% CI)** | **Odds Ratio**  **(95% CI)** |
| Pre-pandemic trend | 1.03 (1.03 to 1.03) | 1.03 (1.02 to 1.04) |
| Shift change at pandemic | 2.23 (2.16 to 2.31) | 1.09 (0.78 to 1.51) |
| Post-pandemic trend | 1.02 (1.01 to 1.02) | 1.10 (1.08 to 1.13) |
| Change in trend from pre to post pandemic | 0.99 (0.99 to 0.99) | 1.07 (1.04 to 1.10) |

### Interrupted time series models of Breaches of the 28-day Cancelled Operation Standard by presence of A&E department using Logistic Regression with robust standard errors

|  | **A&E department** | **No A&E department** |
| --- | --- | --- |
| **Time period** | **Odds Ratio**  **(95% CI)** | **Odds Ratio**  **(95% CI)** |
| Pre-pandemic trend | 1.03 (1.02 to 1.03) | 1.03 (1.01 to 1.05) |
| Shift change at pandemic | 2.23 (1.84 to 2.72)) | 1.09 (0.72 to 1.64) |
| Post-pandemic trend | 1.02 (1.00 to 1.03) | 1.10 (1.08 to 1.13) |
| Change in trend from pre to post pandemic | 0.99 (0.97 to 1.01) | 1.07 (1.05 to 1.09) |
